# Blood-based tumor biomarkers: integrating biological layers and analytical strategies for precision cancer detection

**DOI:** 10.3389/fmed.2026.1815382

**Published:** 2026-09-08

**Authors:** Yi Zhang, Zhen Wang

**Affiliations:** 1 College of Life Sciences, Zhejiang University, Hangzhou, Zhejiang, China; 2 Laboratory Medicine Center, Allergy Center, Department of Transfusion Medicine, Zhejiang Provincial People’s Hospital (Affiliated People’s Hospital), Hangzhou Medical College, Hangzhou, China

**Keywords:** circulating tumor biomarkers, liquid biopsy, microfluidic, molecular diagnostics, regulatory signatures

## Abstract

Blood-based cancer biomarkers promise minimally invasive, longitudinal monitoring, yet their clinical utility varies profoundly by analyte class, disease stage, and analytical methodology. This Review critically evaluates six circulating biomarker classes, such as CTCs, ctDNA, cfDNA methylation, exosomes, circulating miRNAs, and tumor-educated platelets—moving beyond a descriptive catalog to a comparative analysis of their performance characteristics. We find that the dominant challenge is no longer signal detection per se, but rather signal interpretation in the presence of biological confounders like clonal hematopoiesis and pre-analytical variability. We argue that a disciplined pairing of a biomarker’s biological origin with the physico-chemical principles of its detection platform is the primary determinant of assay robustness. The Review concludes by outlining a pragmatic framework for integrating orthogonal biomarker readouts, emphasizing that how we combine signals is a more pressing question than how many markers we can measure.

## 1 Introduction

Early and accurate cancer detection remains one of the most persistent and structurally unresolved challenges in modern oncology (1, 2). Despite continuous advances in imaging technologies and molecular pathology, clinical diagnosis is still dominated by tissue biopsy, a strategy intrinsically constrained by invasiveness, spatial sampling bias, and temporal rigidity (3, 4). These constraints increasingly conflict with the biological nature of tumorigenesis, which unfolds as a dynamic, multistep process driven by clonal evolution, genomic instability, epigenetic reprogramming, and sustained interactions between malignant cells and the host microenvironment (5–7). As a result, static tissue-based assessments frequently fail to capture early malignant transformation, intratumoral heterogeneity, and adaptive resistance processes that ultimately determine disease trajectory and therapeutic outcome (8).

This mismatch is most evident during precancerous and early-stage disease, where tumor burden is minimal and lesions are often anatomically inaccessible or radiologically occult (9). Even in advanced cancer, repeated tissue sampling is rarely feasible owing to procedural risk, patient burden, and limited representativeness of localized biopsies. These limitations have catalyzed a paradigm shift toward minimally invasive diagnostic strategies capable of system-wide and longitudinal interrogation, rather than reliance on isolated snapshots of local tissue architecture (10).

Liquid biopsy has emerged as a conceptual and technological response to these unmet needs (11). Among available biofluids, peripheral blood occupies a central role due to its accessibility, reproducibility, and capacity to integrate molecular information released from both primary tumors and metastatic sites (12). A growing body of clinical and translational evidence demonstrates that blood harbors a diverse spectrum of tumor-associated components reflecting essential hallmarks of cancer, including genetic alterations, epigenetic dysregulation, immune modulation, and metastatic competence (13). Importantly, blood sampling naturally enables serial collection, allowing molecular changes to be tracked over time and often detected before overt clinical or radiographic progression.

The clinical value of blood-based cancer detection depends on two interdependent factors: the biological relevance of circulating biomarkers and the analytical performance of the technologies used to interrogate them (14). Tumor-derived signals in blood are frequently present at extremely low abundance, particularly during early disease or following curative-intent therapy, and are embedded within a vast background of host-derived material (15). Consequently, biomarker selection must be grounded in mechanistic links to tumor biology, while detection strategies must achieve high sensitivity, specificity, and robustness under clinically realistic conditions.

**Figure 1:**
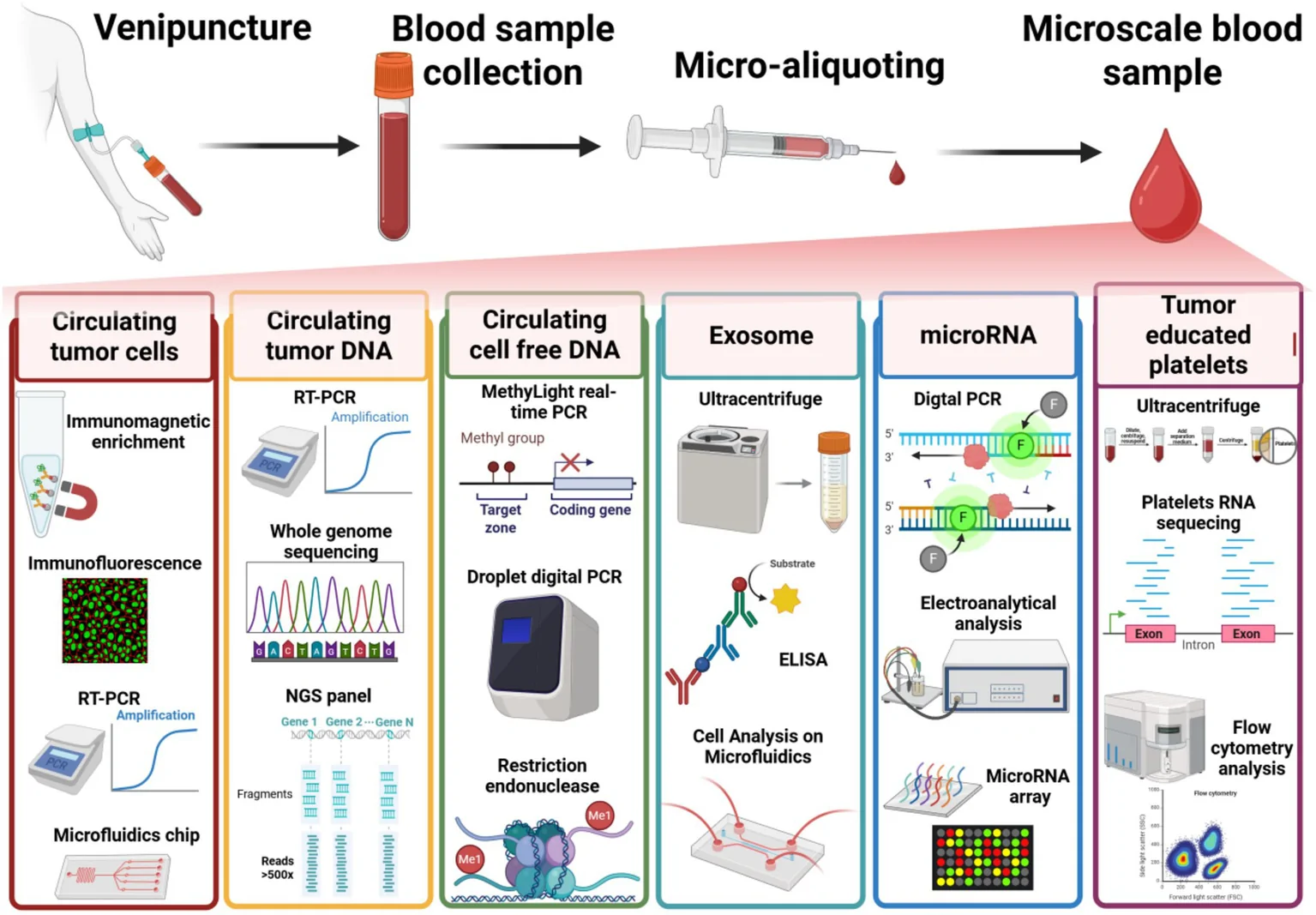
Integrated blood-based liquid biopsy framework for precision cancer detection and POCT analysis. Schematic overview of the multistep workflow, from microscale blood collection through biomarker-specific enrichment and detection, to integrated multi-analyte readout. The framework highlights how complementary circulating biomarkers—CTCs, ctDNA, cfDNA methylation, exosomes, miRNAs, and TEPs—can be combined within a single analytical pipeline to support sensitive, longitudinal, and clinically actionable cancer surveillance.

Over the past decade, multiple classes of blood-derived biomarkers have emerged as informative and complementary. Circulating tumor cells (CTCs) provide cellular-level insight into tumor dissemination and phenotypic plasticity. Circulating tumor DNA (ctDNA) encodes tumor-specific genomic alterations and reflects clonal dynamics. Cell-free DNA (cfDNA) methylation patterns capture epigenetic states associated with early transformation. Extracellular vesicles, particularly exosomes, convey functionally organized molecular cargo that mediates tumor–host communication (16). Circulating microRNAs report pathway-level regulatory states, whereas tumor-educated platelets (TEPs) integrate tumor-induced reprogramming of host platelets into a systemic biosensing platform (Figure 1).

Importantly, these circulating analytes should not be viewed as interchangeable surrogates of tumour presence. Rather, each biomarker class occupies a distinct diagnostic niche: CTCs are particularly informative for dissemination phenotype and metastatic competence. ctDNA is best suited to clonal tracking and minimal residual disease assessment, cfDNA methylation provides structured epigenetic information for early detection and tissue-of-origin inference; exosomes and circulating miRNAs capture functional and regulatory states that may precede overt genomic readouts; and tumour-educated platelets offer a systems-level representation of tumour–host coupling (17). This distinction is essential not only for biomarker interpretation, but also for rational selection of analytical methods and clinical deployment pathways. Together, these complementary analytes define a multidimensional blood-based representation of cancer biology (18). Advances in analytical chemistry, microfluidics, nucleic-acid amplification, and high-throughput sequencing have progressively reduced sample volume requirements, increased analytical throughput, and enhanced sensitivity for ultra-low-abundance targets. These developments are accelerating a transition from centralized laboratory testing toward point-of-care testing (POCT) platforms capable of rapid, multiplexed, and clinically actionable analysis. This Review systematically examines major classes of circulating tumor-associated biomarkers alongside the forefront analytical approaches used for their detection, with particular emphasis on mechanistic relevance, methodological rationale, and translational feasibility.

To ensure a balanced and reproducible synthesis, we defined our review scope around the analytical and clinical validation of blood-based biomarkers. We prioritized inclusion of (i) large-cohort, prospective clinical studies, (ii) systematic reviews and meta-analyses with defined statistical endpoints, and (iii) head-to-head technology comparisons. We largely excluded case reports and small, single-center feasibility studies lacking independent validation. While no formal meta-analytic ranking was performed, we qualitatively weighted studies based on cohort size, sample handling protocol transparency, and validation rigor (independent test set vs. cross-validation). This structured approach allows us to distinguish biomarker candidates with definitive clinical evidence from those still in early translational exploration, a critical distinction often blurred in narrative reviews.

## 2 Integrated blood-based biomarkers and analytical strategies for precision cancer detection, monitoring, and early warning

### 2.1 Pre-analytical variables as a determinant of interpretability in blood-based cancer assays

Before biomarker-specific performance can be meaningfully interpreted, it is necessary to recognize that the analytical reliability of blood-based cancer assays is strongly conditioned by pre-analytical handling. This issue is not merely procedural (19). Because many circulating tumour-associated signals exist at extremely low abundance and are embedded in a complex background of host-derived material, even modest deviations in sample collection, processing, and storage can alter the biological composition of the specimen and distort downstream readouts (20). Accordingly, pre-analytical standardization should be regarded as a prerequisite for cross-study comparability and clinical implementation rather than as a secondary technical detail.

Several variables are particularly consequential. The choice of blood collection tube and anticoagulant can influence cellular integrity, nucleic-acid stability, and platelet activation. For ctDNA and cfDNA methylation analyses, delayed processing may lead to leukocyte lysis and release of background genomic DNA, thereby diluting tumour-derived signals and compromising low-frequency variant or methylation detection. In miRNA and exosome studies, storage time, freeze–thaw cycles, and temperature fluctuations may affect vesicle integrity, RNA recovery, and apparent biomarker abundance (21). For tumour-educated platelets, sample handling is especially critical because ex vivo platelet activation can substantially reshape both transcriptomic and phenotypic profiles, thereby confounding interpretation of tumour-associated signatures.

A systematic study demonstrated that a processing delay from 2 h to 6 h at room temperature resulted in a median 2.3-fold increase in background wild-type cfDNA concentration from leukocyte lysis, effectively halving the detectable tumor fraction and compromising the sensitivity of low-VAF mutation calling (22). Similarly, variability in centrifugation protocols can introduce a coefficient of variation (CV) exceeding 35% in residual platelet counts, which is a primary source of contamination in tumor-educated platelet RNA-seq studies. These are not marginal effects; they represent quantifiable sources of variance that must be modeled and standardized to define fit-for-purpose operating procedure tolerances. For these reasons, future progress in blood-based cancer diagnostics will depend not only on improved analytical sensitivity, but also on tighter harmonization of sampling workflows, stabilization chemistries, storage conditions, and processing timelines. In practical terms, technologies with strong clinical potential will be those that are not only analytically powerful, but also resilient to pre-analytical variability under routine operating conditions.

### 2.2 Circulating tumor cells: cellular tracers of early dissemination and metastatic potential

CTCs’ clinical significance is therefore inseparable from mechanism: intravasation is favoured by epithelial–mesenchymal plasticity, altered cell–matrix adhesions, vascular remodelling, and immune escape, while survival in shear stress and oxidative pressure selects for resilient subclones. In this sense, CTCs should be regarded as a selective readout of tumour evolution, not merely a proxy for tumour presence (23).

**Figure 2:**
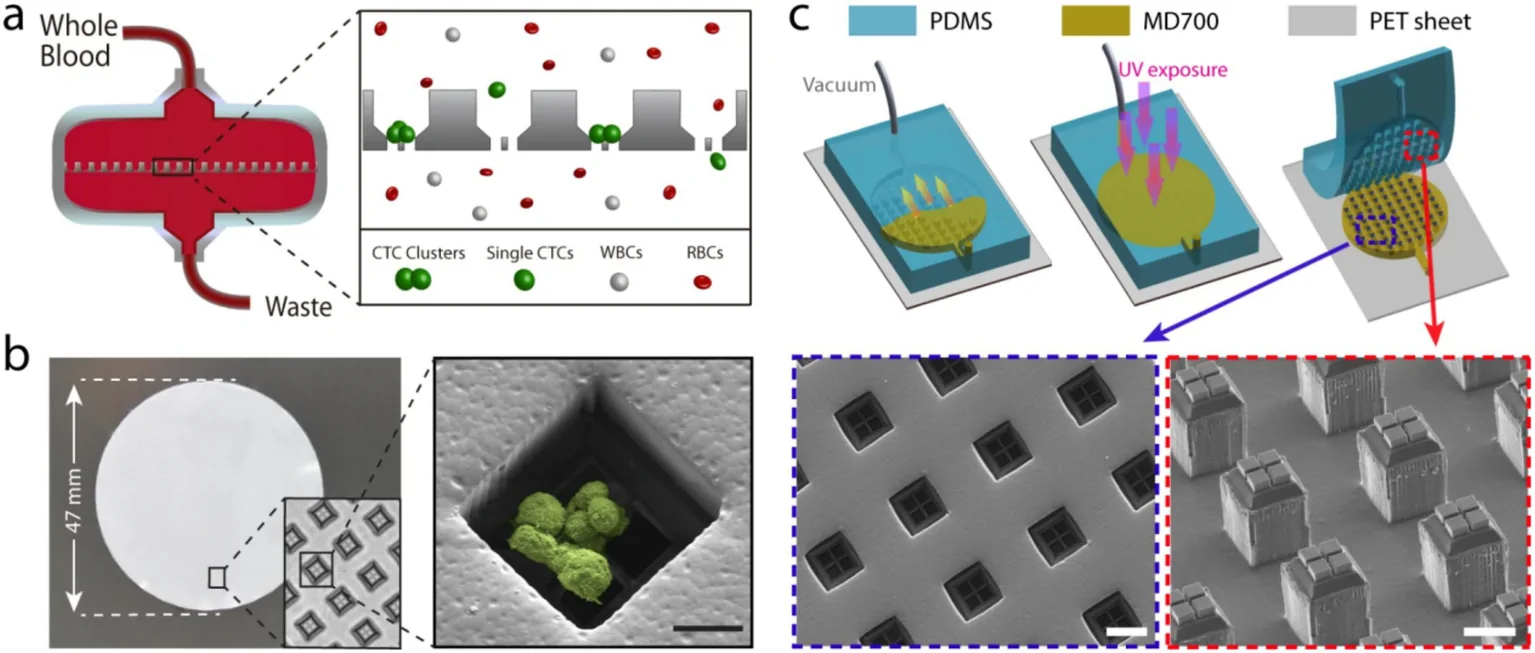
Microfluidic isolation of circulating tumor cell clusters using the Cluster-Wells platform. **(A)** Schematic illustration of the Cluster-Wells working principle. Single blood cells pass through the micromesh openings, whereas multicellular circulating tumor cell (CTC) clusters are physically retained on the basis of their size and multicellular geometry. **(B)** Photograph and enlarged view of the Cluster-Wells membrane device, showing individual microwells designed for cluster capture and a representative captured tumor-cell cluster. **(C)** Schematic representation of the microfabrication process and representative micrographs of the patterned microwell structures. The label-free architecture enables high-throughput isolation of intact CTC clusters while reducing dependence on variable epithelial surface markers. Adapted from Boya et al. (26), under the Creative Commons Attribution 4.0 International License (CC BY 4.0).

This mechanistic framing explains why CTC enumeration alone is often insufficient; it is the composition of the CTC compartment - single cells versus clusters, epithelial-like versus mesenchymal-like states, and the preservation of viable aggregates—that more directly maps to progression risk (23). Microfluidic devices designed for cluster retention have therefore gained prominence because they translate a biological property (aggregate cohesion) into a physical separation rule. The Cluster-Wells architecture (Figure 2) exemplifies this logic: clusters are retained by microwells incorporating a micromesh that allows blood cells to pass while arresting multicellular aggregates, effectively converting cluster geometry into a deterministic capture principle. Such label-free capture reduces dependence on variable epithelial markers and can better accommodate phenotypic drift under therapy (24).

Downstream, nanomagnetic bead enrichment and immunofluorescence imaging remain valuable for scalable workflows, enabling multiplexed phenotyping and morphological adjudication. RT-PCR-based readouts provide sensitive confirmation of tumour-associated transcripts when cell counts are low, but their interpretability benefits from orthogonal validation due to the risk of background expression in non-malignant compartments (25). Conceptually, the most durable trajectory is toward integrated systems in which enrichment preserves viable cellular states while readouts resolve heterogeneity in a clinically interpretable manner—a direction in which microfluidics, automated imaging, and compact molecular assays increasingly converge (26). If CTCs capture the cellular phenotype of dissemination, the next analyte—ctDNA—provides an orthogonal readout: a genomic record of clonal dynamics that is more readily standardised for longitudinal tracking, yet indifferent to the phenotypic state of the cells that shed it.

### 2.3 Circulating tumor DNA: a genomic continuum spanning burden, residual disease, and adaptation

If CTCs operationalise dissemination at the cellular level, circulating tumor DNA (ctDNA) encodes tumour evolution in a fragmentary yet analytically tractable form (27). Released predominantly through apoptosis and necrosis—with additional contributions from active secretion—ctDNA carries somatic point mutations, indels, and structural alterations that collectively mirror clonal architecture and its temporal reshaping under therapeutic pressure. Importantly, ctDNA is not merely a “molecular shadow” of the tumour: its concentration, allelic fraction, and fragmentation landscape are shaped by tumour turnover, vascular access, and clearance kinetics, which together determine when ctDNA becomes detectable and how faithfully it reports evolving disease states (28). This mechanistic coupling has sharpened the clinical rationale for ctDNA testing across the last 5 years, particularly in settings where standard radiography is intrinsically insensitive to microscopic disease.

The strongest translational pull has come from minimal residual disease (MRD). A landmark prospective study in colorectal cancer demonstrated that ctDNA-MRD detection, at variant allele fractions (VAFs) as low as 0.01% achieved by tumor-informed NGS, preceded radiographic recurrence by a median of 8.7 months, with a sensitivity of 88% at specificity exceeding 95% (29, 30). In contrast, in breast cancer, the sensitivity of post-surgical MRD detection drops substantially, often requiring deeper sequencing depths (>100,000x) to overcome lower circulating tumor fractions (31). This highlights a critical performance gap: while an LOD of 0.01% VAF is sufficient for some gastrointestinal cancers, it may still be an order of magnitude too high for cancers with low DNA shedding, a quantitative benchmark often absent from conceptual discussions.

Method selection in ctDNA analysis is best framed as a trade-space between breadth, depth, and turnaround, with each axis mapping to a distinct clinical question. RT-PCR and allele-specific qPCR remain pragmatic for narrowly defined tasks, such as tracking a known hotspot mutation at high frequency, owing to their operational simplicity and rapid turnaround (32). However, their fixed target scope is poorly aligned with clonal diversification, where emergent subclones may not carry the sentinel variant being monitored. Whole-genome sequencing (WGS) expands breadth and enables genome-wide copy-number and fragmentomic inference, but depth requirements, cost, and computational burden can restrict routine deployment in clinical pathways that demand high sensitivity at low tumour fractions (33). Targeted next-generation sequencing (NGS) has therefore become the translational centre of gravity, particularly when paired with unique molecular identifiers, duplex consensus strategies, and statistical error modelling that suppress technical artefacts to the level required for MRD calling (33).

**Figure 3:**
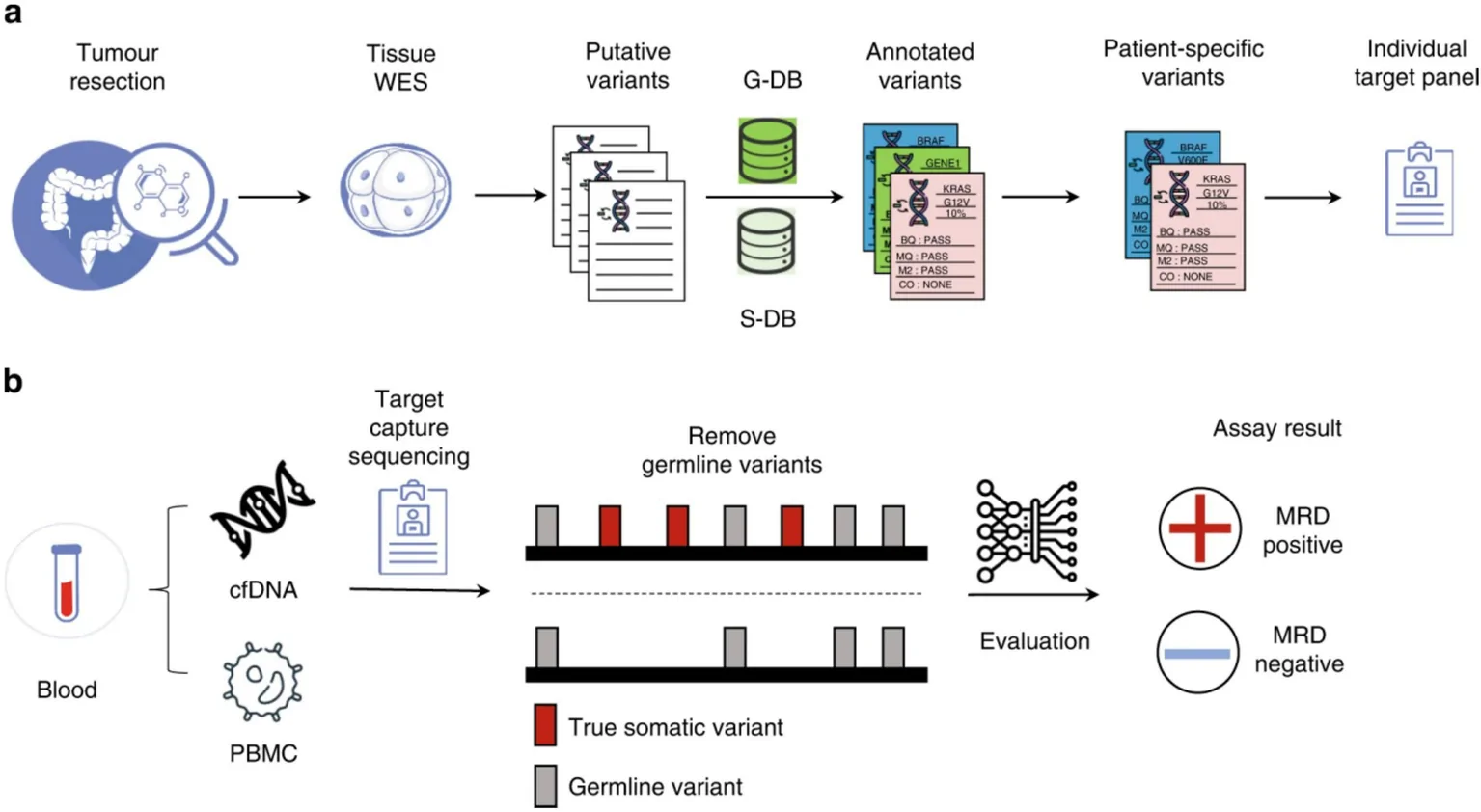
Tumor-informed targeted next-generation sequencing workflow for ultrasensitive detection of circulating tumor DNA and minimal residual disease. **(A)** Patient-specific panel design begins with tumor resection and whole-exome sequencing (WES), followed by variant identification and annotation, removal of likely germline variants, and selection of patient-specific somatic mutations for individualized target-panel construction. **(B)** Peripheral blood is processed to obtain cell-free DNA (cfDNA) and peripheral blood mononuclear cells (PBMCs). Target-capture sequencing and matched background filtering are subsequently used to identify true somatic variants and classify samples as minimal residual disease (MRD)-positive or MRD-negative. This tumor-informed strategy increases analytical sensitivity while reducing interference from germline variants and other background signals. Adapted from Ryoo et al. (43), under the Creative Commons Attribution 4.0 International License (CC BY 4.0).

Tumour-informed workflows (Figure 3) provide a mechanistic rationale for this sensitivity (30). Patient-specific somatic variants are first identified from tumour tissue—often via whole-exome sequencing—and curated against germline resources and, where available, matched leukocyte DNA to mitigate confounding by clonal haematopoiesis. A personalised capture panel is then designed for deep plasma sequencing, enriching a defined set of informative loci while enabling stringent error suppression. The AlphaLiquid®Detect scheme illustrates this pipeline explicitly, including variant annotation, germline filtering, and rational target selection before capture-based NGS is applied to plasma for MRD detection. By anchoring detection to tumour-private mutations and enforcing systematic background control, tumour-informed designs align analytical capability with the biological question that matters most in the adjuvant setting: whether residual malignant clones persist at levels below conventional detectability, and whether they are expanding under selective pressure (34).

Among currently available blood-based approaches, ctDNA analysis is arguably the closest to broad clinical adoption, particularly in the context of molecular residual disease assessment and therapy-response monitoring. Nevertheless, its performance remains constrained by low tumour fraction, background error accumulation, and biological confounders such as clonal haematopoiesis, all of which become especially consequential when variant calls are interpreted near the analytical detection limit. For this reason, continued progress will depend not only on deeper sequencing and improved error suppression, but also on clearer reporting standards, tighter pre-analytical control, and clinically validated thresholds that distinguish residual disease from background molecular noise (35).

A further, frequently underestimated source of biological noise in ctDNA analysis is clonal hematopoiesis of indeterminate potential (CHIP). This age-related phenomenon introduces somatic mutations—most commonly in DNMT3A, TET2 and ASXL1—into hematopoietic cells, and the variant allele frequencies (VAFs) of these CHIP-derived signals in plasma typically range between 0.1% and over 10%, directly overlapping the detection window used for minimal residual disease calling. A recent study quantified this overlap across 16,812 advanced cancer patients: 42.3% of liquid biopsy profiles harbored at least one CHIP variant among reportable clinical genes, and in patients aged 80 years or older, the median proportion of CHIP-attributable variants reached 50%. A specific study demonstrated that paired tumor-WBC sequencing increased the proportion of patients eligible for plasma MRD tracking from 89% to 100% and revealed CHIP variants in 27% of a localized colon cancer cohort (36, 37). These data underscore that, without parallel WBC sequencing, CHIP variants can masquerade as tumor-derived signals—most concerningly in genes such as TP53, ATM and BRCA1/2, and matched leukocyte analysis is not merely a technical refinement but a prerequisite for clinically interpretable liquid biopsy results (37).

Regulatory agencies (FDA, EMA) increasingly accept MRD as an enrichment strategy, yet the bar for a full surrogate endpoint is higher: a formal meta-level demonstration that a therapeutic effect on MRD capture reliably translates into a treatment effect on survival (38). Without that evidence, claims of “clinical readiness” overstate what has been shown (39). While ctDNA tracks the genome, early-stage disease often leaves a sparse mutational footprint. Epigenetic alterations, particularly aberrant DNA methylation, can be more abundant at low tumour burden—and remain chemically stable in cfDNA fragments—making them a complementary early detection signal.

### 2.4 cfDNA epigenetic signatures as sensitive indicators of t1umor initiation, progression, and clinical monitoring

In contrast, epigenetic dysregulation at CpG-rich regulatory regions is tightly coupled to lineage identity and remains chemically stable within cfDNA fragments (40). These properties confer two clinically valuable advantages: first, methylation patterns can amplify biological contrast even when mutant alleles are rare; second, the tissue-informative structure of methylation landscapes provides a route to infer tumour origin, offering information that is orthogonal to mutation-centric ctDNA profiling (41).

**Figure 4:**
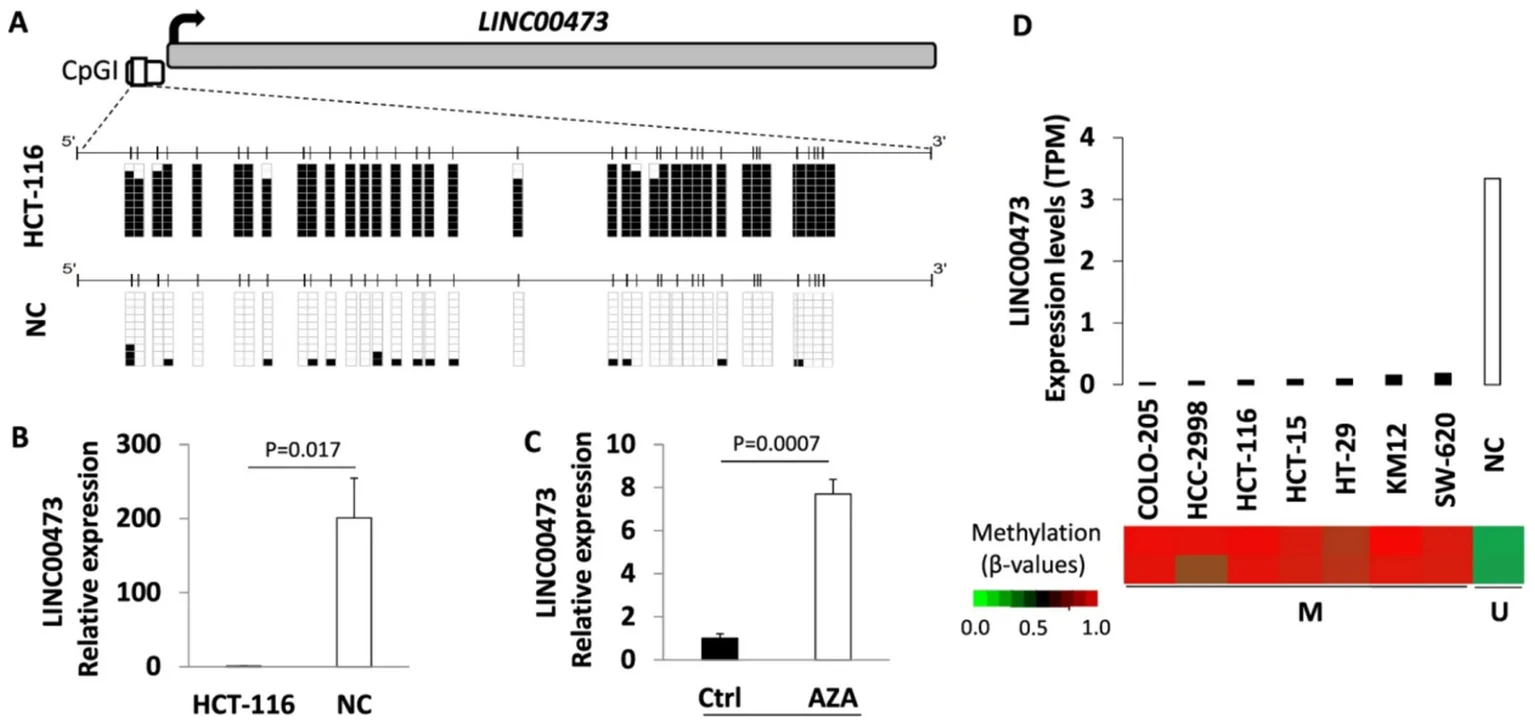
Epigenetic silencing of LINC00473 through promoter hypermethylation in colorectal cancer. **(A)** Bisulfite genomic sequencing analysis of the LINC00473 promoter CpG island in the colorectal cancer cell line HCT-116 and normal colorectal tissue (NC), demonstrating extensive promoter methylation in HCT-116 cells. **(B)** LINC00473 expression is reduced in HCT-116 cells compared with normal colorectal tissue, consistent with methylation-associated transcriptional silencing. **(C)** Treatment of HCT-116 cells with the DNA-demethylating agent 5-aza-2′-deoxycytidine (AZA) restores LINC00473 expression, supporting a relationship between promoter methylation and transcriptional repression. **(D)** Integrated methylation and expression analysis across colorectal cancer cell lines and normal colon mucosa demonstrates an inverse relationship between LINC00473 promoter methylation and gene expression. Together, these findings illustrate the biological basis for exploiting cancer-associated DNA methylation as a circulating epigenetic biomarker. Adapted from Ruiz-Bañobre et al. (79), under the Creative Commons Attribution 4.0 International License (CC BY 4.0).

Methylation-specific PCR (MSP) and quantitative MSP operationalise this biology through bisulfite conversion–enabled locus discrimination (42). Bisulfite treatment converts unmethylated cytosines to uracils while leaving 5-methylcytosines protected, allowing primers and probes to selectively amplify methylated targets with high analytical sensitivity. Figure 4, illustrates this principle by showing the relationship between LINC00473 promoter hypermethylation and transcriptional silencing in colorectal cancer, providing a mechanistic basis for the use of cancer-associated methylation signatures as circulating epigenetic biomarkers (43). In particular, the schematic underscores a staged pathway in which candidate methylation markers are first prioritised based on biological and statistical criteria, then transferred into a standardised qMSP workflow compatible with routine plasma processing, and finally tested within a structured, prospective clinical design that reflects real diagnostic contexts rather than retrospective convenience (44). This progression is not merely procedural; it directly addresses the major vulnerabilities of methylation assays—locus selection bias, pre-analytical variability, and overfitting—by embedding assay development within controlled sample handling, predefined performance endpoints, and external validation across centres (45).

Droplet digital PCR extends the same biochemical discrimination into a partitioned, molecule-counting regime, improving quantification at ultra-low allele fractions and enabling more stable longitudinal comparisons (46). Restriction endonuclease–based strategies provide a complementary route to methylation interrogation without full bisulfite conversion, potentially preserving cfDNA integrity and mitigating conversion-associated loss, which can be limiting when input is low (47).

Taken together, cfDNA methylation methodologies offer a plausible and increasingly evidence-supported route to reduce reliance on large blood volumes while improving early-stage sensitivity, precisely because they target structured epigenetic information that persists when point mutations are rare or stochastically sampled (48).

Relative to mutation-based ctDNA assays, cfDNA methylation approaches are particularly attractive in early-stage settings, where epigenetic disruption may be more abundant and more tissue-informative than somatic mutation signals. Their principal limitations arise from assay design itself, including locus-selection bias, bisulfite-associated DNA loss, and uncertainty regarding the transferability of marker panels across cohorts, tumour types, and processing workflows. Even so, methylation-based assays occupy an increasingly important translational niche because they address one of the most difficult diagnostic problems in liquid biopsy: how to achieve sensitivity when tumour-derived genomic signal is weak but biologically structured epigenetic information remains accessible (49).

Both ctDNA and cfDNA methylation interrogate free-floating DNA fragments, but they inevitably lose information on the membrane-organised signalling complexes that tumour cells actively secrete. Exosomes preserve this functional architecture, shifting the analysis from circulating fragments to circulating vehicles.

### 2.5 Exosomes: functional carriers whose heterogeneity becomes analytically actionable

Methodologically, ultracentrifugation remains the most widely adopted isolation strategy and continues to serve as a biological reference standard (50). By exploiting size and buoyant density differences, differential and density-gradient ultracentrifugation enables enrichment of vesicles within the canonical exosomal range and has been instrumental in defining exosome composition and function (51). However, its time-intensive workflow, dependence on specialised equipment, and limited scalability constrain routine clinical deployment. Immunoassay-based approaches, particularly ELISA, offer a more clinically tractable alternative by capturing exosomes via conserved tetraspanins (CD9, CD63, CD81) or tumour-associated antigens. Extensions of this strategy to immune-relevant cargo, such as exosomal PD-L1, have directly linked vesicle profiling to therapeutic response assessment, albeit within a predefined target scope (52).

**Figure 5:**
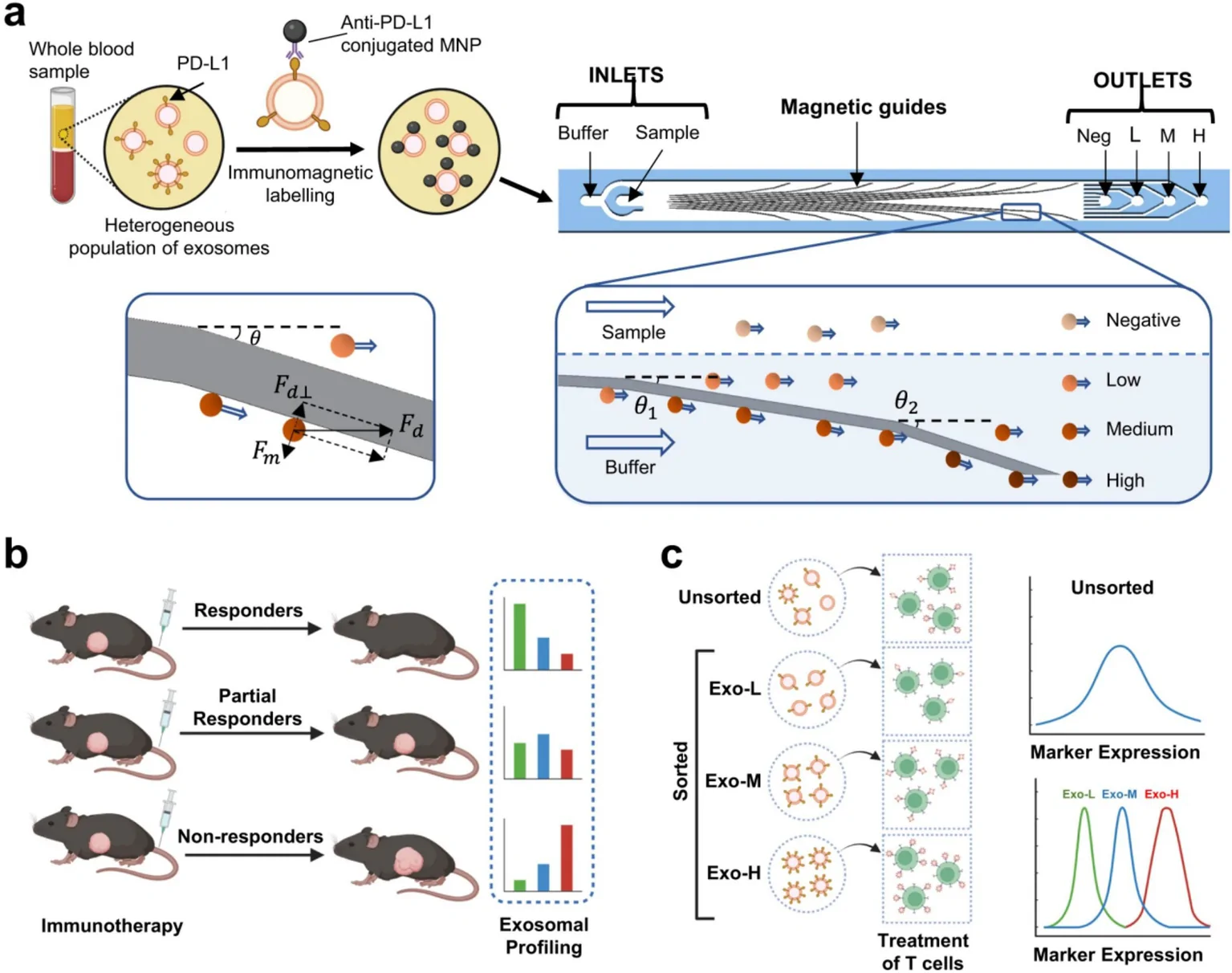
NanoEPIC-based immunomagnetic profiling and sorting of PD-L1-positive small extracellular vesicle subpopulations. **(A)** Schematic illustration of the NanoEPIC platform, in which small extracellular vesicles (sEVs) are labeled with anti-PD-L1-conjugated magnetic nanoparticles and introduced into a microfluidic channel containing magnetic guides. Magnetically induced deflection enables separation of vesicle subpopulations according to PD-L1-associated magnetic labeling. **(B)** Application of NanoEPIC-based exosomal PD-L1 profiling to experimental and immunotherapy-related samples, illustrating differences in vesicle phenotypes associated with treatment response. **(C)** Fractionation of sEV subpopulations with different PD-L1 levels and downstream functional analysis of their interactions with T cells, demonstrating how vesicle heterogeneity can be translated into phenotypically and functionally informative readouts. Adapted and assembled from Chen et al. (59), under the Creative Commons Attribution 4.0 International License (CC BY 4.0).

Microfluidic-based exosome sorting has therefore emerged as a pivotal technological advance. As illustrated in Figure 5, microfluidic platforms integrate size-selective transport and immunoaffinity capture within precisely engineered channels, enabling efficient isolation and phenotypic resolution of exosome subpopulations from microlitre-scale samples (53). The NanoEPIC-type architectures highlighted in the figure exemplify how vesicle heterogeneity—rather than bulk abundance—can be rendered analytically actionable, allowing selective enrichment of tumour-associated or immune-modulatory exosomes. By minimising sample loss, shortening processing time, and facilitating seamless coupling with downstream molecular readouts, such systems convert exosome biology into a format compatible with automation and decentralised testing (54).

Collectively, ultracentrifugation, ELISA, and microfluidic sorting define a methodological continuum that balances biological fidelity, analytical specificity, and translational feasibility (55). Continued refinement and standardisation of microfluidic exosome technologies are expected to play a central role in advancing exosome-based assays from mechanistic investigation toward clinically actionable liquid biopsy and precision cancer diagnostics (56).

A major strength of exosome-based analysis lies in its ability to preserve functional and phenotypic information that is often diluted or lost in cell-free nucleic acid assays. However, this same complexity introduces methodological challenges, including incomplete separation from non-vesicular contaminants, subpopulation heterogeneity, and limited harmonization of reference standards across platforms. In practical terms, exosome assays may be most immediately valuable in contexts where immune state, signalling phenotype, or vesicle-associated therapeutic markers are more informative than mutation status alone. Their longer-term impact will likely depend on whether microfluidic and affinity-based systems can deliver not only higher sensitivity, but also reproducible and clinically interpretable subtype-resolved measurements (57). Exosomes carry diverse molecular cargo, but their miRNA content merits particular attention. Unlike vesicle-bound proteins, miRNAs are stable in circulation even outside vesicles. They are bound to Argonaute proteins or other RNA-binding partners, providing a regulatory level signal that complements both genomic and proteomic readouts.

### 2.6 Circulating microRNAs: stable regulatory signals read out across scales

As short non-coding RNAs that post-transcriptionally regulate gene expression, miRNAs are deeply embedded in the molecular circuitry governing tumor initiation, progression, and adaptation (58, 59). Dysregulated miRNA networks contribute to oncogenic transformation by modulating cell-cycle control, apoptosis, epithelial–mesenchymal transition, immune evasion, and metabolic reprogramming (60). Over the past 5 years, large-scale profiling and mechanistic studies have consistently demonstrated that tumor-associated miRNAs are not only altered at very early stages of malignant transformation but are also actively released into circulation in highly stable forms, either bound to RNA-binding proteins or encapsulated within extracellular vesicles (54). This intrinsic stability, together with their pathway-level regulatory roles, establishes circulating miRNAs as reliable integrators of tumor dynamics across disease stages.

From a clinical standpoint, miRNA detection addresses gaps that remain unresolved by other circulating biomarkers. Whereas ctDNA primarily reflects genetic alterations, miRNAs report functional regulatory states and signaling activity. Distinct miRNA signatures have been shown to discriminate benign lesions from early malignancy and to stratify progressive disease even under low tumor burden, highlighting their value for early detection and longitudinal risk assessment (61). These attributes make miRNA analysis a necessary component of precision oncology and blood-based point-of-care testing frameworks.

**Figure 6:**
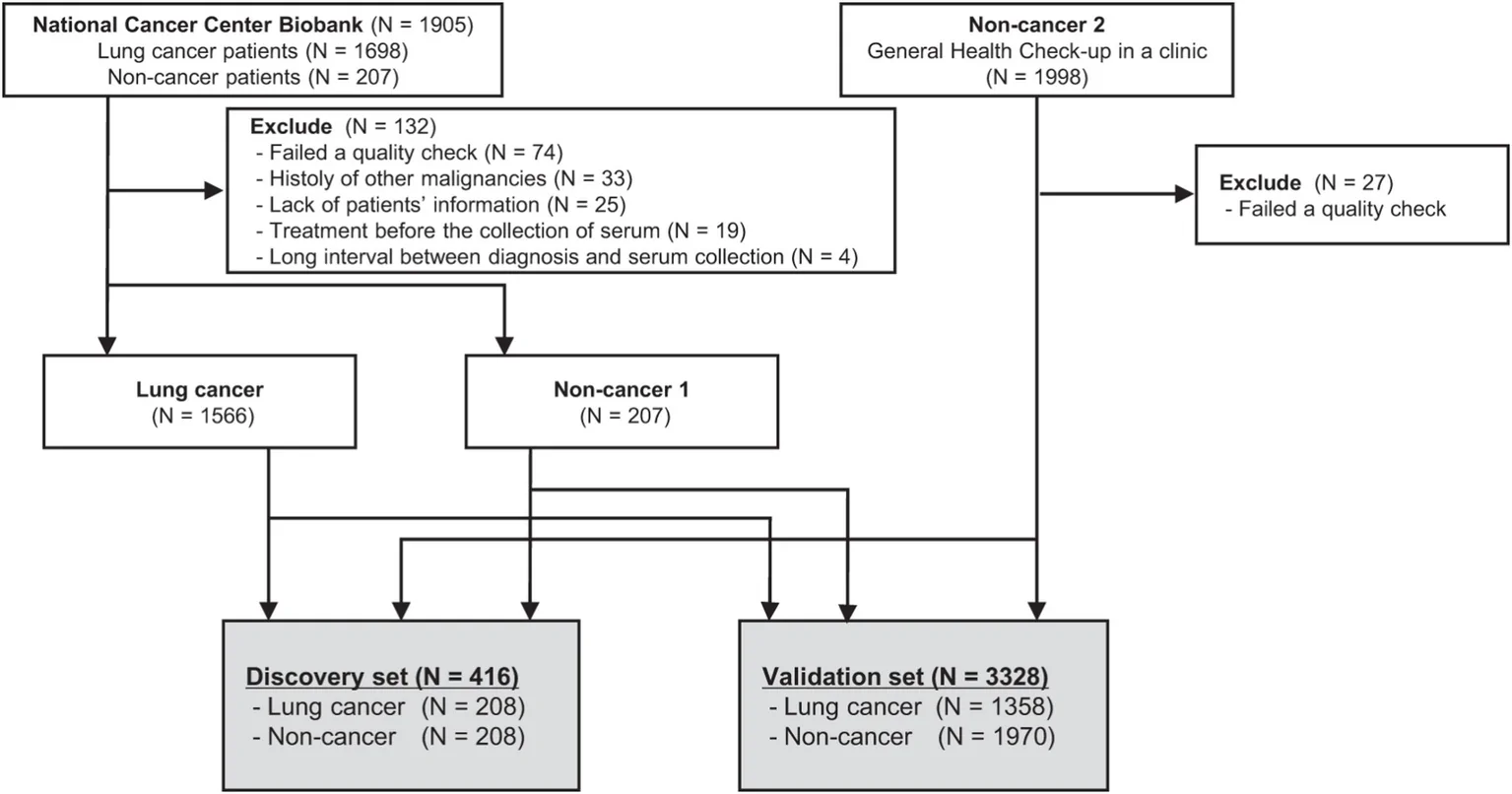
Cohort design for circulating miRNA-based lung cancer biomarker discovery and validation. Flow diagram illustrating patient selection, exclusion criteria, and allocation of lung cancer and non-cancer participants derived from the National Cancer Center Biobank and a general health-screening population. Eligible samples were allocated to discovery and validation cohorts for systematic circulating miRNA profiling and development of a diagnostic model. The large-scale, independently validated cohort design illustrates the translational pathway from high-dimensional miRNA profiling to clinically applicable circulating biomarker signatures. Adapted from Asakura et al. (80), under the Creative Commons Attribution 4.0 International License (CC BY 4.0).

Technologically, digital PCR (dPCR) enables absolute quantification of low-abundance miRNAs through reaction partitioning and direct molecule counting, supporting sensitive monitoring of treatment-induced fluctuations and minimal residual disease–associated signatures (62). Electrochemical detection strategies translate miRNA–probe hybridization into electrical readouts, offering rapid response, low sample consumption, and compatibility with miniaturized systems (63). Hybridization-based probe arrays remain indispensable for discovery and validation. As illustrated in Figure 6, array-based workflows integrate standardized serum processing, high-density probe hybridization, and computational modeling to derive disease-specific miRNA signatures from large cohorts. Such designs emphasize reproducibility, cross-cohort validation, and biological interpretability, enabling systematic compression of high-dimensional miRNA data into clinically tractable classifiers (64). Although less suited for rapid bedside deployment, array platforms provide the evidentiary backbone upon which targeted dPCR or electrochemical assays can be rationally constructed.

Collectively, dPCR, electrochemical sensing, and hybridization arrays define a complementary methodological spectrum for miRNA detection (65). Continued advances in sensitivity, normalization, and platform integration are expected to further solidify circulating miRNAs as clinically actionable biomarkers for early cancer detection, dynamic monitoring, and precision-guided intervention.

From a translational standpoint, miRNA platforms may need to be viewed as functionally distinct rather than directly competing. High-dimensional arrays are best positioned for discovery, classifier training, and biological validation across large cohorts, whereas dPCR and electrochemical systems are better aligned with targeted quantification and deployment in constrained clinical settings. Despite their stability, circulating miRNAs face a formidable specificity crisis that is often underappreciated. The “normalization problem” is not just a technical nuisance; it is a fundamental confounder. Common reference controls like miR-16 or U6 snRNA are themselves dysregulated in various cancers and subject to hemolysis-induced fluctuations, rendering the very yardstick unstable. Furthermore, the assumption that tumor-derived miRNAs dominate the circulating pool is frequently violated. A large fraction of the circulating miRNome, for instance miR-21, is elevated in inflammation, fibrosis, and other non-malignant conditions. This results in cross-study inconsistencies wherein a signature validated in a carefully controlled cohort completely fails to replicate in a real-world population with high comorbidity burden. Thus, the critical failure mode of miRNA biomarkers is not analytical sensitivity, but biological specificity, and addressing it demands a shift from single-miRNA candidate-level metrics to pathway-informed, multi-miRNA expression scores validated explicitly against diverse non-cancer controls (66). All biomarkers discussed so far originate, directly or indirectly, from the tumour mass. By contrast, TEPs reflect a fundamentally different information source: the host’s systemic response to tumour presence. This shift from tumour-derived to host-reprogrammed signals opens a complementary dimension in liquid biopsy.

### 2.7 Platelet reprogramming by tumors as a systemic signal for cancer detection and translational liquid biopsy

From a clinical perspective, TEP analysis addresses several limitations inherent to other blood-based biomarkers. Platelets are abundant, stable, and readily isolated from peripheral blood, enabling reliable signal acquisition even when tumor burden is extremely low (67). Notably, tumor-induced platelet reprogramming often precedes detectable changes in ctDNA levels or radiographic findings, underscoring the value of TEPs for early detection and risk stratification (68). Moreover, platelet phenotypes reflect not only tumor genotype but also tumor–host interactions, including inflammatory signaling, coagulation pathways, and immune escape mechanisms. This integrative nature allows TEPs to capture biological dimensions that are poorly represented by nucleic acid–only assays, reinforcing their importance within comprehensive precision oncology frameworks.

Methodologically, differential centrifugation-based platelet isolation remains a foundational step for TEP analysis (69). Differential centrifugation protocols enable enrichment of highly purified platelet fractions while minimizing contamination from leukocytes and plasma-derived nucleic acids. Although robust and widely adopted, this approach is labor-intensive and sensitive to pre-analytical variation, particularly platelet activation, necessitating careful standardization and motivating the development of streamlined workflows (70).

At the molecular level, platelet RNA sequencing has emerged as a powerful strategy for decoding TEP signatures. High-throughput transcriptomic profiling reveals tumor-induced changes in mRNA abundance, alternative splicing, and non-coding RNA content, enabling discrimination between cancer and non-cancer states and, in some cases, inference of tumor origin (71). Machine-learning models trained on platelet transcriptomes have achieved high diagnostic accuracy across multiple tumor types, highlighting strong translational potential, albeit currently best suited to centralized testing environments (72).

**Figure 7:**
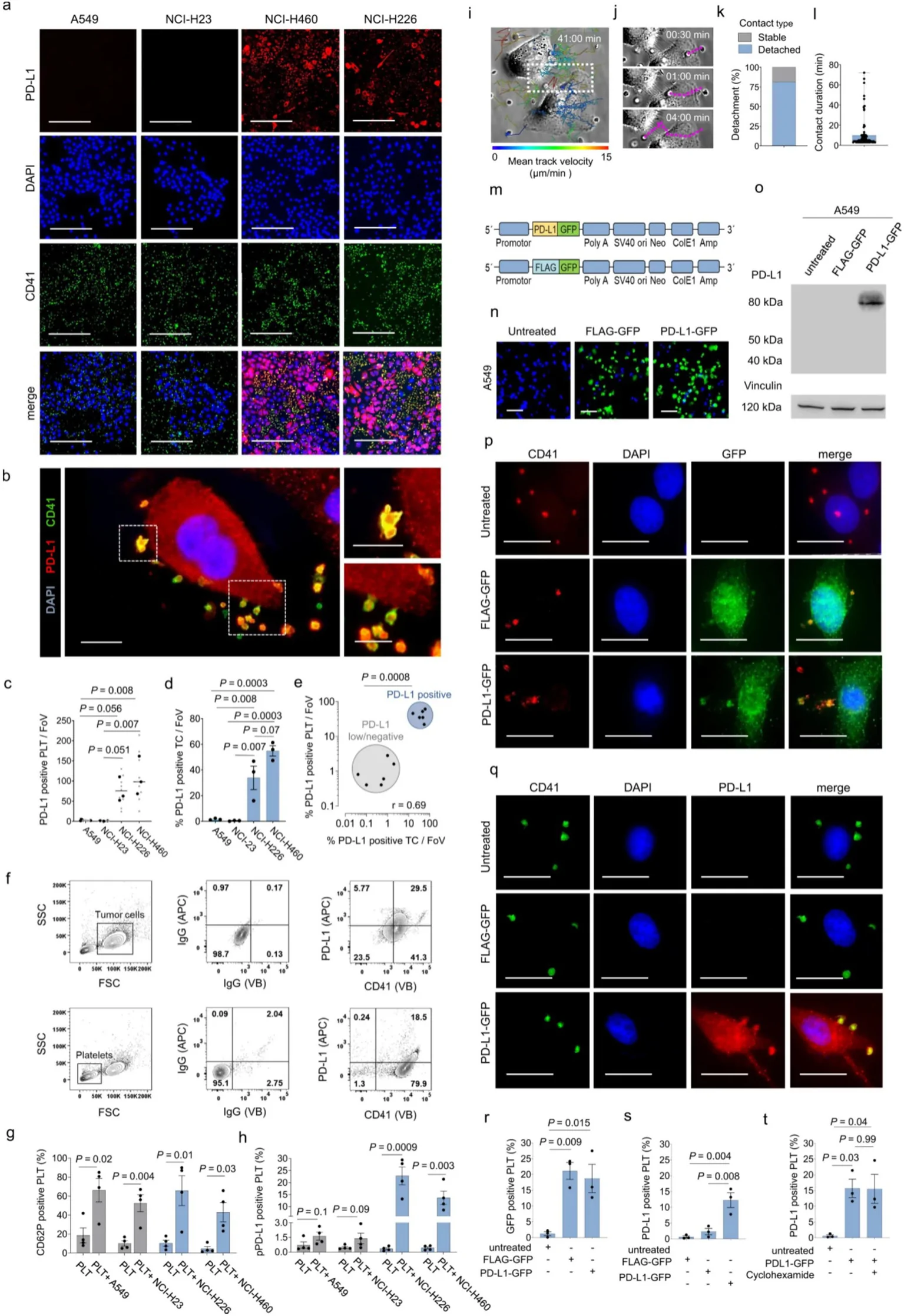
Experimental characterization of platelet PD-L1 as a tumor-associated immune phenotype in lung cancer. **(A-F)** Experimental characterization of PD-L1 expression and its association with platelet and tumor-cell compartments using immunofluorescence microscopy and flow cytometry, including analysis of CD41-positive platelet-associated signals. **(G-L)** Genetic and biochemical experiments examining the origin, transfer, and regulation of platelet-associated PD-L1, supported by representative flow-cytometric and immunoblot analyses. **(M-T)** Quantitative and functional analyses of platelet-tumor interactions and PD-L1-positive platelet populations under different experimental conditions, illustrating how tumor-associated immune phenotypes can be transferred to and detected on circulating platelets. Collectively, these experiments establish platelet PD-L1 as a measurable surrogate of tumor-associated PD-L1 biology and provide the mechanistic basis for flow-cytometric assessment of tumor-educated platelet phenotypes. Adapted and assembled from Hinterleitner et al. (74), under the Creative Commons Attribution 4.0 International License (CC BY 4.0).

Flow cytometry provides a complementary, phenotype-oriented approach that bridges biological insight and clinical practicality. By interrogating platelet activation markers, immune-related surface receptors, and tumor-associated antigens, flow-based assays enable rapid, single-cell–level assessment of functional reprogramming (73). Figure 7 exemplifies this strategy by illustrating standardized gating of platelets and quantification of PD-L1–positive subpopulations, directly linking platelet phenotypes to tumor immune status and therapeutic response. Advances in multiparameter flow cytometry and harmonized antibody panels have further improved reproducibility and throughput, positioning this modality as a realistic pathway toward clinically deployable TEP assays (74).

Collectively, differential centrifugation, platelet RNA sequencing, and flow cytometry form a convergent methodological framework that balances biological depth with translational feasibility. Continued refinement of these approaches, particularly flow-based phenotyping integrated with other circulating biomarkers, is expected to advance TEP-based assays from proof-of-concept toward clinically actionable tools for early detection, dynamic monitoring, and precision cancer management. TEPs complete the spectrum of blood-based analytes surveyed in this Review, moving the measurement locus from tumour output to host reprogramming. The question now is not which single marker performs best, but how to couple complementary biological layers into integrated, clinically resilient assays—a question we address in the concluding synthesis.

## 3 Frontier science and clinical pragmatics as co-drivers of blood-based cancer POCT

A central lesson emerging from recent liquid biopsy research is that blood is not simply a convenient substitute for tissue; it is an information-bearing compartment that continuously integrates tumour evolution with host response. The clinical motivation is straightforward—conventional diagnosis remains constrained by invasiveness, sampling bias and temporal rigidity—but the scientific imperative is deeper: tumorigenesis is a distributed, multistep process shaped by clonal competition, epigenetic state transitions, immune editing and microenvironmental feedback (75). Capturing such dynamics demands biomarkers that are mechanistically anchored and technologies that remain reliable under the reality of ultra-low abundance and high biological background.

Within this study, the six circulating factors discussed in this Review should be interpreted as layer-specific reporters rather than interchangeable “signals” (76). Each factor resolves a different dimension of malignancy—cellular dissemination, genomic variation, epigenetic lineage memory, intercellular communication, regulatory network activity, and systemic host reprogramming—while each analytical strategy is best judged by how precisely it translates that biological property into a robust measurement. In frontier research, this alignment enables mechanistic insight; in clinical practice, it governs reproducibility, interpretability and actionability.

CTCs occupy the most direct mechanistic position because they instantiate dissemination as a cellular phenotype. Their value lies not merely in enumeration but in preserving the state space of metastatic competence—single cells versus clusters, epithelial-like versus plastic states, viability and stress-adaptation. This is why microfluidic architectures that exploit physical determinants, rather than single-marker dependence, represent more than engineering elegance: they reduce selection bias introduced by marker loss and enable downstream analyses that remain faithful to the biology of phenotypic drift. In practice, this makes CTC-based workflows well suited to prognostic stratification and to the early identification of aggressive dissemination trajectories, especially when morphology and multiparametric phenotyping are integrated into a standardized pipeline. ctDNA, by contrast, is a genomic trace whose clinical power comes from repeatability and quantitative dynamics. Importantly, ctDNA detectability is governed by tumour turnover, vascular access and clearance kinetics, so it encodes both clonal composition and the physiological conditions that release tumour fragments into circulation. This dual dependency has made ctDNA especially impactful in minimal residual disease (MRD), where imaging is intrinsically underpowered and where molecular positivity can serve as an early surrogate of recurrence risk. Technically, the key frontier has been error suppression and patient-specific targeting: tumour-informed NGS workflows translate the individuality of somatic variation into sensitivity by designing personalised panels after systematic germline filtering and variant curation (Figure 3). The clinical implication is not simply “better detection,” but the possibility of re-framing adjuvant management as a risk-adaptive intervention space, provided specificity is protected against confounders such as clonal haematopoiesis and pre-analytical noise.

cfDNA methylation addresses a different bottleneck: early tumorigenesis may not present abundant mutations, yet epigenetic dysregulation can be pronounced and lineage-informative. Methylation thus offers a structured signal that can remain measurable at low tumour fraction. MSP/qMSP, ddPCR adaptations and enzyme-based assays form a spectrum in which the underlying chemistry (bisulfite discrimination or methylation-sensitive digestion) is used to convert epigenetic state into an amplification-compatible readout. Figure 4 is particularly instructive because it embodies how epigenetic marker discovery can be disciplined into clinically meaningful validation: prospective, multi-centre study design and workflow compatibility with routine plasma handling explicitly reduce the risk of overfitting and improve reproducibility across realistic clinical conditions.

In practice, this positions methylation as a credible route for early detection and tissue-of-origin inference, complementing mutation-centric ctDNA tests. Exosomes expand liquid biopsy beyond molecular fragments by preserving cargo organisation and membrane identity—properties that directly reflect tumour–host communication. Their relevance is amplified by function: exosomal programmes participate in immune modulation, metabolic rewiring and niche formation, so the measurable object is not merely “presence” but a functional phenotype. Traditional ultracentrifugation remains biologically foundational, but clinical translation increasingly favours methods that are faster, lower-loss and compatible with small volumes. ELISA adds target specificity, yet can obscure vesicle heterogeneity. Microfluidic immunomagnetic sorting, as captured in Figure 5, resolves this limitation by making subpopulation structure measurable: selective enrichment of tumour-associated or immune-modulatory exosomes turns heterogeneity into a diagnostic advantage rather than an analytical nuisance.

Clinically, such systems are compelling because they bridge mechanistic interpretability with device-level integration and automation. Circulating miRNAs represent regulatory-state biomarkers. Their distinguishing feature is that they report pathway activity and network rewiring, not discrete genomic events; their stability in circulation enables robust sampling across stages (77). In frontier work, the principal challenge is moving from high-dimensional profiling to clinically stable classifiers. Figure 6 illustrates this translational path: standardized serum processing coupled to high-throughput hybridization arrays and model construction yields signatures that can be distilled into smaller panels suitable for targeted platforms.

Practically, arrays anchor discovery and validation, whereas dPCR and electrochemical sensing offer routes to absolute quantification and portable testing, especially when integrated with microfluidics or catalytic signal amplification. Tumor-educated platelets (TEPs) shift the locus of information from tumour output to host reprogramming. This distinction matters: platelets integrate tumour signalling with coagulation, inflammation and immune escape, providing a systemic readout that nucleic-acid–only assays may underrepresent. From a clinical viewpoint, abundance and ease of isolation strengthen robustness at low tumour burden. From a technological perspective, platelet RNA-seq remains the most information-rich discovery tool, but flow cytometry provides a pragmatic bridge to deployment. Figure 7 highlights why: standardized gating strategies and PD-L1–linked phenotyping operationalise tumour–immune coupling into an assay format that is familiar to clinical laboratories and potentially compatible with high-throughput workflows.

Taken together, these biomarkers and methods outline a clear direction for both frontier science and clinical practice: the most durable strategies are those that co-design analyte choice with measurement physics/chemistry, embed error control at the level required by ultra-low abundance, and maintain interpretability across longitudinal sampling. In the short term, this means rationally integrated panels rather than single-analyte dependence; in the longer term, it implies an evolution toward microscale, multiplexed, and automated POCT systems that can support early warning and adaptive intervention without sacrificing analytical rigour (Table 1).

**Table 1:** Biological dimensions, analytical readouts, translational niches, and key limitations of major blood-based tumor biomarkers.

Circulating factor	Biological information encoded	Key analytical readouts	Representative detection methods	Frontier research relevance	Clinical application value	Key limitations/analytical challenges
Circulating tumor cells (CTCs)	Cellular dissemination, metastatic competence, phenotypic plasticity	Cell counts, clusters, EMT/stem-like phenotypes, viability	Microfluidic isolation, magnetic enrichment, immuno-fluorescence imaging, RT-PCR	Mechanistic insight into metastasis and therapy resistance	Prognostic stratification and dissemination risk assessment	Rarity, capture bias, phenotypic drift, inter-platform variability
Circulating tumor DNA (ctDNA)	Tumor-derived genomic alterations and clonal evolution	Variant allele fraction, mutation spectrum, MRD status, copy-number changes	Allele-specific PCR, targeted NGS, whole-genome sequencing	Real-time tracking of clonal dynamics and resistance emergence	Minimal residual disease detection and recurrence prediction	Low tumour fraction, CHIP interference, error suppression demands, pre-analytical background
cfDNA methylation	Epigenetic dysregulation and tissue lineage identity	Locus-specific methylation indices, tissue-of-origin classifiers	qMSP, ddPCR, methylation-sensitive restriction enzyme assays	Early tumorigenesis and epigenetic classifier development	Early cancer detection and screening applications	Locus-selection bias, conversion-associated DNA loss, inter-cohort transferability
Exosomes	Tumor–host communication and immune-modulatory signaling	Vesicle subpopulations, surface proteins (e.g., PD-L1), cargo profiles	Ultracentrifugation, ELISA, microfluidic immunomagnetic sorting	Functional signaling phenotypes and vesicle heterogeneity analysis	Immunotherapy stratification and response monitoring	Isolation purity, subtype heterogeneity, limited reference standardization
Circulating microRNAs	Post-transcriptional regulatory network activity	miRNA signatures, panel abundance, longitudinal dynamics	Hybridization arrays, digital PCR, electrochemical sensing	Systems-level regulation and biomarker signature discovery	Early-stage stratification and longitudinal disease monitoring	Normalization uncertainty, non-tumour background sources, variable disease specificity
Tumor-educated platelets (TEPs)	Host reprogramming integrating immune and coagulation pathways	Transcriptomic shifts, activation markers, PD-L1-positive platelets	Platelet RNA sequencing, flow cytometry, differential centrifugation	Systemic tumor–host interaction mapping	Early warning signals and immune response prediction	Platelet activation during handling, inflammatory confounding, workflow harmonization

A particularly important direction emerging from recent studies is the development of multi-analyte liquid biopsy platforms that combine orthogonal biological dimensions rather than maximizing a single signal type. This strategy is conceptually attractive because no individual biomarker class fully resolves the complexity of tumour evolution: ctDNA captures clonal dynamics but not functional phenotype; methylation profiles improve early detection but may not reflect current therapeutic adaptation; exosomes and miRNAs report signalling state but may lack tumour exclusivity; and platelet-based signals reflect host response but not tumour genotype in a direct sense. Integrated panels therefore offer a route to improved sensitivity and interpretability by linking tumour-intrinsic and host-derived information within the same analytical framework. The key methodological challenge, therefore, lies not in combining markers per se, but in defining task-specific, mathematically sound combinations. Simple heuristic “positivity in any one marker” approaches inevitably inflate false-positive rates. Consequently, computational integration frameworks have become essential. For instance, regularized logistic regression or random forest classifiers are now frequently used to integrate ctDNA mutations with cfDNA methylation signatures, using cross-validation to penalize model complexity and reduce overfitting. More advanced multi-view learning models, such as Bayesian networks, have demonstrated the ability to fuse heterogeneous data types—including miRNA-seq counts, ctDNA fragmentomic features, and exosomal protein markers—into a unified probabilistic risk score. Crucially, such integration requires overcoming batch effects and platform-specific biases, a practical reality that demands pre-processing steps like ComBat harmonization, which is rarely discussed but is a prerequisite for any clinically deployable, multi-analyte panel (78) (Table 2).

**Table 2:** Hierarchy of clinical evidence for blood-based cancer biomarkers.

Biomarker class	Clinical application	Level of evidence	Key study designs
ctDNA	MRD detection (colorectal)	High	Multiple prospective, interventional phase III trials
cfDNA methylation	Multi-cancer early detection	Moderate	Large, prospective case–control studies (e.g., CCGA); lack of mortality reduction data
Circulating miRNAs	Diagnosis (HCC, lung)	Moderate-low	Majority retrospective, single studies; meta-analyses show high heterogeneity across cohorts
Exosomes	Immunotherapy response	Low	Mostly small, single-center cohorts; assay standardization lacking
TEPs	Pan-cancer diagnosis	Low (exploratory)	High accuracy in cross-validation but lack of independent, locked-model prospective validation

## 4 Conclusion and outlook

Blood-based tumour biomarkers have progressed from proof-of-concept indicators to analytically sophisticated readouts capable of resolving distinct layers of cancer biology. As synthesised throughout this Review, circulating cells, nucleic acids, vesicles and host-reprogrammed components do not merely provide redundant information; rather, they encode complementary biological dimensions that collectively describe tumour initiation, progression and therapeutic adaptation. The critical challenge now is not further demonstration of feasibility, but strategic advancement toward technologies that deepen biological resolution while remaining compatible with clinical constraints.

From a frontier research perspective, several directions warrant focused exploration. First, physical-property–guided microfluidic systems are likely to evolve beyond enrichment toward functional interrogation. Devices that integrate deformability, electrical properties and transient adhesion dynamics may enable real-time phenotyping of CTCs and exosome subpopulations without reliance on predefined markers, thereby capturing phenotypic plasticity that underlies metastatic competence and treatment resistance. Second, epigenome-resolved cfDNA analysis remains underexploited. Beyond methylation density alone, emerging approaches that combine methylation, fragmentation topology and nucleosome positioning could substantially improve early-stage sensitivity and tissue-of-origin assignment, particularly when mutation burden is minimal. Third, platelet-based sensing represents an underdeveloped systems-level biomarker. Integrating platelet phenotyping with spatial transcriptomics or proteomic readouts may provide new insight into tumour–host coupling, immune escape and coagulation-driven niche formation.

In parallel, translational priorities must emphasise robustness, standardisation and interpretability. Technologies most likely to succeed clinically are those that tolerate low input volumes, minimise pre-analytical sensitivity and deliver outputs that map cleanly onto decision thresholds. In this context, tumour-informed targeted NGS is well positioned for minimal residual disease monitoring, where sensitivity and longitudinal consistency outweigh breadth. Flow cytometry–based assays, particularly for tumour-educated platelets and immune-relevant exosomal markers, offer a pragmatic balance between biological specificity and laboratory familiarity, facilitating deployment in routine clinical settings. Likewise, digital PCR and electrochemical platforms provide scalable routes for absolute quantification of defined nucleic acid targets, lending themselves to decentralised testing and, ultimately, point-of-care implementation.

Looking forward, the greatest gains are likely to arise from rationally coupled biomarker strategies rather than single-analyte optimisation. Combining genomic ctDNA readouts with epigenetic cfDNA signatures can align clonal tracking with lineage context; pairing exosomal immune markers with platelet phenotyping may link tumour signalling to systemic immune status; integrating miRNA regulatory signatures with mutation-based assays could reconcile functional state with genetic substrate. Such coupling demands not only technical compatibility but conceptual integration—analytical pipelines designed around specific clinical questions, rather than maximal data generation.

At present, the most clinically mature blood-based strategies are those that answer relatively focused questions under well-defined analytical constraints, such as ctDNA-guided molecular residual disease assessment or targeted monitoring of known genomic alterations. Other modalities—including exosome subtype analysis, multi-marker CTC phenotyping, and TEP-based systemic readouts—are progressing rapidly but remain more dependent on platform harmonization, cohort-scale validation, and assay simplification before broader adoption becomes realistic. A key priority for the field is therefore to distinguish translational promise from immediate deployability, and to advance each technology according to the clinical question it is best suited to address. Ultimately, the trajectory of blood-based cancer detection will be shaped by convergence: convergence of biological layers, of analytical modalities, and of research and clinical workflows. As technologies mature, the emphasis should shift from sensitivity alone toward predictive precision, where dynamic blood-derived signals inform not only whether cancer is present, but how it is likely to evolve and respond to intervention. Achieving this goal will require continued interdisciplinary collaboration, rigorous validation in prospective cohorts, and thoughtful translation of frontier innovation into clinically resilient platforms.
